# Animal-Assisted Interventions for Children with Advanced Cancer: Child and Parent Perceptions

**DOI:** 10.1089/pmr.2021.0039

**Published:** 2021-11-17

**Authors:** Brittany A. Cowfer, Terrah Foster Akard, Mary Jo Gilmer

**Affiliations:** ^1^ Department of Pediatric Hematology/Oncology, Vanderbilt University Medical Center and Monroe Carell Jr. Children's Hospital at Vanderbilt, Nashville, Tennessee, USA.; ^2^ Vanderbilt University School of Nursing, Nashville, Tennessee, USA.

**Keywords:** animal-assisted interventions, childhood cancer, pediatric cancer, pediatric palliative care

## Abstract

***Background:*** The burden of relapsed/refractory childhood cancer takes an immense toll on ill children and their caregivers, jeopardizing quality of life. Animal-assisted interventions (AAIs) have shown promising benefits for children with chronic conditions and their families. Little is known about child and caregiver perspectives on AAI participation for children with advanced cancer.

***Objective:*** To explore perspectives of children with advanced cancer and their caregivers on experiences with AAIs.

***Design:*** Cross-sectional qualitative design.

***Setting/Subjects:*** Participants were children (*n* = 9) aged 5 to 17 years with relapsed or refractory cancer and their parents (*n* = 12) from one academic children's hospital in the southeastern United States. Participants completed approximately weekly 15-minute AAI sessions with a trained dog and handler during oncology clinic visits or hospitalizations for up to 12 weeks.

***Measures:*** Semistructured interviews were carried out after completion of each family's final AAI session to assess child and parent perceptions of AAIs. Qualitative content analysis identified themes.

***Results:*** Five themes emerged: (1) positive aspects, (2) negative aspects, (3) preferred changes, (4) pet ownership, and (5) value of the study. Twenty (95%) participants shared positive aspects of AAIs. The only negative aspect reported was too little time with the dog.

***Conclusion:*** Children with advanced cancer and their parents perceive AAIs as desirable with few requested changes. Further studies are needed to fully evaluate impact of AAIs.

ClinicalTrials.gov Identifier: NCT03765099.

## Introduction

In 2021, an estimated 15,590 children and adolescents will be diagnosed with cancer, and 1780 will die of the disease in the United States.^1^ Despite advances in treatment, childhood cancer remains the leading cause of disease-related death in U.S. children.^1^ Cancer treatment dramatically alters everyday childhood routines, resulting in extended school absences and isolation from peers due to hospitalizations, surgeries, frequent clinic visits, and risk of infection. Children newly diagnosed with cancer have described feeling shocked and overwhelmed, as well as worrying about their families.^2^ Both cancer and its treatment can result in numerous physical symptoms including pain, fatigue, nausea, and difficulty sleeping, which are often distressing for children and parents.^3–5^

Cancer relapse often triggers negative memories from initial treatment, heightening distress, especially in light of more limited treatment options and a more tangible fear of death.^6^ Treatment for relapse is often more intensive than the initial diagnosis, requiring more frequent clinic appointments and hospitalizations. Children with advanced cancer have reported high physical and psychological symptom burden and rated their symptoms as highly distressing, whereas those nearing end of life have reported even greater prevalence of most symptoms.^7^ A number of distressing symptoms, including dry mouth, pain, fatigue, vomiting, insomnia, anorexia, worry, sadness, irritability, and difficulty concentrating, have been shown to reduce health-related quality of life in children with advanced cancer.^8^ Families have frequently experienced increased uncertainty and anxiety after disease relapse, often striving to maintain a sense of control and normalcy.^6,9,10^

Animal-assisted interventions (AAIs), or goal-oriented interventions (including animal-assisted therapy) that intentionally utilize animals to support achievement of individual therapeutic goals and improve health and wellness, have been shown to benefit children in the health care environment.^11^ Benefits include reduced procedural anxiety and distress,^12^ pain reduction,^14–17^ improved sense of well-being during hospitalization,^18^ and decreased anxiety.^19^ Hospitalized children who received AAIs reported feelings of joy and calmness, more positive memories from hospitalization, and a sense that time passed more quickly.^18^ In parents of children with cancer, AAIs decreased parenting stress^17^ and anxiety.^17^ AAIs have also been shown to improve pain,^17^ stress,^17^ depressive symptoms,^17^ and quality of life^21^ in children with cancer. Furthermore, studies have shown that AAI is safe without increased infectious risk to children.^21^

Despite numerous demonstrated benefits of AAIs, there are limited data regarding efficacy of AAIs to mitigate suffering and improve quality of life in the vulnerable population of children with relapsed or refractory cancer. Although prior studies have shown that children with cancer and their parents perceived AAIs as beneficial and were satisfied with the intervention,^21–24^ few studies have explored child or parent perceptions of the AAI experience. Given the greater burden of relapsed/refractory childhood cancer and potential for greater positive impact, it is critical to explore family member perspectives on the experience of AAIs in this unique and vulnerable population. Thus, the aim of this qualitative study was to explore child and caregiver perceptions of an AAI.

## Methods

### Design

This was a cross-sectional qualitative study, which was a part of a larger randomized controlled trial conducted at a single institution in the southeastern United States, evaluating the efficacy of AAI sessions for health-related quality of life for children with advanced cancer and stress and anxiety in their primary parent caregivers.^25^ Data collection took place from July 2019 through April 2021. This article reports qualitative findings from participants who received the AAI and participated in an interview at study end.

### Participants

Children 3 to 17 years of age with advanced (relapsed or refractory) cancer and their primary parent caregivers were recruited to participate in the study. Caregivers were 18 years of age and older and were the parent(s) who primarily accompanied the child to their clinic visits and hospitalizations. Caregivers and children were excluded if they were unable to understand and speak English, had cognitive impairment, or had a self-reported fear of or allergy to dogs.

After obtaining institutional review board approval, children with advanced cancer at the institution were screened for eligibility. The research team obtained approval from the child's medical team before approach. A trained research team member approached children and caregivers to introduce the study and gauge interest during a routine clinic visit and proceeded with informed consent and assent for interested caregivers and children.

Participant demographic characteristics are described in Table 1. Child median age was nine years. About half of children and parents self-identified as white. Most parents were female and married. Annual family income was widely distributed, with participants in every category ranging from $15,000 to >$100,000.

**Table 1.** Sample Demographic and Clinical Characteristics (*N* = 21)

	*N*	Median (range)
Child with cancer		
Age, years	9	9 (5–17)
		*n* (%)
Diagnosis		
Leukemia/lymphoma	9	4 (44)
Extracranial solid tumor		2 (22)
CNS tumor		3 (33)
Gender		
Male	9	5 (56)
Female		4 (44)
Race		
White/Caucasian	9	5 (56)
African American		1 (11)
Other		3 (33)
Caregiver		
Age range, years		
18–25	11	1 (9)
26–35		5 (45)
36–46		5 (45)
Gender		
Male	12	3 (25)
Female		9 (75)
Race		
White/Caucasian	10	5 (50)
African American		2 (20)
Other		3 (30)
Current marital status		
Single	11	3 (27)
Married		8 (73)
Current annual family income		
$15,000–$24,999 per year	11	2 (18)
$25,000–$34,999 per year		2 (18)
$35,000–$49,999 per year		3 (27)
$50,000–$74,999 per year		1 (9)
$75,000–$99,999 per year		2 (18)
$100,000 or more per year		1 (9)

### AAI

The intervention consisted of approximately weekly visits from a registered canine and handler who were in good standing with their animal-assisted therapy organization, Pet Partners. Visits were ∼15 minutes in duration and occurred during the child's routine clinic visits or hospitalizations over a period of up to 12 weeks as often as weekly. Each child had between 2 and 11 AAI sessions (median 6) over the 12-week period. During each visit, participants were allowed to pick activities to engage in with the dog, such as petting the dog, talking to the dog, or having the dog do tricks and feeding the dog treats. All researchers adhered to all recommended precautions, including careful hand sanitization before and after AAIs and screening of animal handlers and research team members for symptoms of possible infection before participation in data collection. To prevent the spread of infection, AAI visits were deferred if a child was on contact, droplet, or airborne precautions due to confirmed or suspected infection. However, neutropenia without concern for infection did not preclude the child from participating in an AAI session.

### Data collection

Caregivers completed a family demographic form within REDCap^26^ (a secure online program for research design and data collection) at baseline. At the conclusion of each child's final AAI visit, researchers interviewed each parent–child dyad, utilizing a semistructured interview guide. Researchers developed the interview questions based on review of the literature of satisfaction surveys and parent comments after 12 weeks of AAIs for children newly diagnosed with cancer.^20^ Three clinical experts reviewed the initial draft of questions and made suggestions to solicit additional feedback and edit wording to avoid social courtesy bias. The principal investigator (PI) and two trained research assistants served as interviewers. All interviewers had experience in interviewing. Additional training included role play and a review of approach to qualitative interviewing offered by the Vanderbilt Qualitative Core. Researchers asked each parent–child dyad the following semistructured interview questions to learn more about perceptions of AAIs among children with advanced cancer and their parents:

Some families tell us they liked seeing the dog each week. Others maybe not as much. What was it like for you to be a part of this project? Was there anything you didn't like? Are there any changes in the visits you would suggest? Do you have a pet at home, and, if so, do you feel that affected how helpful your animal-assisted interactions were? Why or why not?

Interviews, which lasted between ∼2 and 10 minutes, were audio-recorded and transcribed verbatim. Two children who participated in AAI sessions but did not engage in the interview were excluded from the analysis. One child was too young to participate in the interview and the other was not feeling well enough to answer questions verbally during the interview.

### Data analysis

Two researchers independently analyzed the data from the interviews through qualitative content analysis. Researchers initially read the transcripts multiple times to appreciate the data in aggregate and determined emerging themes. After two researchers independently coded the data, they met to discuss the rationale for code categorization and coding discrepancies and to confirm that data saturation had occurred. Initial inter-rater reliability was 0.92. In the cases of disagreements, a third researcher coded the data to reach consensus.

## Results

Five themes emerged from the data: (1) positive aspects, (2) negative aspects, (3) preferred changes, (4) pet ownership, and (5) value of the study. Themes with counts, frequencies, and exemplar quotes are given in Table 2 and described further hereunder.

**Table 2.** Summary of Qualitative Results

Theme	Definition	Participant (*N* = 21) Counts^a^ *n* (%)	Parents (*N* = 12) Counts^a^ *n* (%)	Child (*N* = 9) Counts^a^ *n* (%)	Exemplar quote
Positive aspects	Enjoyment of intervention; benefits of intervention	20 (95)	11 (92)	10 (100)	“I think it was really great. We loved it. It was very helpful.”
Negative aspects	Dislikes about the intervention	1 (5)	1 (8)	0 (0)	“We spend little time with [name of dog].”
Preferred changes	Requested changes to the intervention	5 (24)	3 (25)	2 (22)	“We wish we had more time with her.”
Pet ownership	Pets at home or lack thereof and impact on helpfulness of the intervention; intervention impact on desire to have a pet	13 (62)	5 (42)	8 (89)	“I think that it probably made me more excited because I don't have a dog at home.”
Value of the study	Reflections on participation in the study and/or need for more dogs to help ill children	7 (33)	6 (50)	1 (11)	“I think [name of child] was very happy to help if it meant more dogs for everyone to get to have while they're here, especially inpatient, because there was a lot of times where he was inpatient and missed his doggie. So, I think we're really happy to help if it means moving towards more dogs for inpatient care.”

^a^ The number (and percentage) of participants who reported each theme.

### Positive aspects

*Positive aspects* of the intervention included general acknowledgment of liking or enjoying AAIs, as well as benefits of the intervention. A nine-year-old male shared, “I liked seeing [name of dog] every week.” The mother of a nine-year-old female shared, “We liked everything about seeing [name of dog]. It made time go faster while we were waiting.”

### Negative aspects

*Negative aspects* of the intervention were those that participants felt to be unfavorable. The mother of a six-year-old male said, “We spend little time with [name of dog].” No other negative aspects were reported by other parents or any children.

### Preferred changes

*Preferred changes* were primarily focused on advocating for more time with the dog for therapeutic benefit. For example, a nine-year-old female said, “I just wish [the visits] could be longer.” The mother of a 16-year-old female shared, “I know you can't always do it, but it would be nice if the dog could come during physical therapy when [name of child] is awake.”

### Pet ownership

*Pet ownership* included discussion of pets at home or lack thereof and perceived effect on helpfulness of the intervention as well as intervention impact on desire for a pet. Most children and parents who did not have a pet felt that the intervention was likely more exciting for them than families with a pet at home because it was novel. A 17-year-old female said, “I think that it probably made me more excited because I don't have a dog at home,” and “we would love to have one like [name of dog].” Children who had pets at home perceived that their experience owning a dog resulted in increased comfort with the intervention, as stated by an 11-year-old female, “I think [having a dog at home] probably made me more comfortable with [name of dog].” Parents echoed these sentiments. The mother of a five-year-old male shared, “Not having an animal at home made it more exciting for him. It was something he looked forward to.”

### Value of the study

*Value of the study* was defined as global reflections on participation in the study and potential benefit of therapy dogs to ill children in general. The father of a seven-year-old male stated, “I think it was a great thing. I am glad that they are looking into it, you know, another thing to help kids because it does give that extra little bit of positivity to the visit, especially during inpatient. I know that our experience with [name of dog] was the only thing that could get him up out of bed. So, I think that if this program does grow, then it is definitely something that could help the kids. And we are very appreciative to be a part of it.”

## Discussion

Although other studies have evaluated the efficacy of AAIs in children with cancer for outcomes of anxiety, stress, pain, quality of life, and intervention satisfaction through a variety of measures, few studies have explored AAI experiences of children with cancer and their parents qualitatively. Our study was the first of its kind to examine child and parent perceptions of a novel AAI delivered to children with relapsed cancer.

Nearly all participants reported positive aspects, such as enjoyment or benefits, from their participation in AAIs. All negative aspects and preferred changes were focused on the topic of desiring additional time with the dog. These findings are consistent with prior research in adult cancer patients, showing high levels of satisfaction with and perceived helpfulness of animal-assisted visits during chemotherapy.^28^ Similar results were found in a study of animal-assisted activities for hospitalized children; survey results indicated that both parents and medical staff felt that child–animal interactions in the hospital were favorable and beneficial.^22^ Furthermore, a prior qualitative study also identified comparable benefits of AAIs for children with cancer, including making children happier and increasing communication with nurses.^24^ Participants in our study did not note changes in communication with the health care team. This may, in part, be due to inclusion of different informants, as the communication findings in the prior study were reported by nurses, whom we did not include in our study.

Half of parents shared their perspective that it is important for more dogs to be allowed in the clinic or hospital to help children. Several parents specifically shared that they were happy to participate as a means of helping fulfill this mission, alluding to a sense of altruism. Altruism has previously been described as an important factor in parents' decision to enroll children with cancer in clinical trials for cancer-directed therapy, although those with the poorest prognoses were less likely to identify altruism as the primary motivation for participation.^29^ Although parents rather than children primarily shared sentiments about helping future patients through their participation, prior studies have also found that adolescents and young adults with cancer identify altruism as important for making meaning of their diagnosis.^30^ Based on parental report of the *value of the study* theme, participation in this supportive care study may have provided an opportunity for families to engage in altruism in a low-stakes manner.

Our study was limited by some variability in conduct of semistructured interviews. Interviews were generally conducted with parent and child together, although individual questions were directed to the parent or child in some interviews, resulting in only one of them sharing their perspective. In addition, some children were too young, did not feel well, or were otherwise occupied and did not respond verbally to interview questions. Some of these limitations are intrinsic to the flexible and prosocial nature of semistructured interviews, as well as the child's age and illness. However, promotion of consistent engagement of both child and parent and allowing them ample opportunities to each share their perspective on all questions may allow for more robust data collection for future studies utilizing this approach.

The COVID-19 pandemic occurred during the data collection period for this study. Given the risks of viral transmission and the vulnerability of the immunocompromised participants, the study was halted in March 2020. AAI visits resumed in September 2020 with modifications, including use of masks, social distancing, and a brief transition to holding AAI visits in the hospital's outdoor space. The disruption in data collection ultimately resulted in children who were enrolled shortly before the pandemic having fewer AAI visits. Thus, the global pandemic likely had an effect on the perspectives of participants over the study period.

The findings from this study have important implications for practice. Although children with relapsed/refractory cancer are frequently more physically and psychologically vulnerable than children with newly diagnosed cancer, parents and ill children perceived AAIs positively and reported no concerns regarding risks of infection, overstimulation, or additional time in the clinic from their experience. Some parents and children requested more time with the dog and felt that AAIs would be beneficial for other children in their position. Thus, despite the fact that many of these children are spending long periods of time in the clinic, providers should advocate for their patients with advanced cancer to receive supportive care services, such as AAIs. Although many of the benefits reported by parents cannot be tangibly measured, it is clear that the experience is meaningful for many families and may ease some of the challenges of the child's treatment.

## Conclusion

Children with advanced cancer and their parents perceive AAIs as beneficial with few requested changes. Additional studies are needed to evaluate overall impact of AAIs.

## Abbreviation Used

AAIs: animal-assisted interventions
